# Acidic, neutral and alkaline forest ponds as a landscape element affecting the biodiversity of freshwater snails

**DOI:** 10.1007/s00114-017-1495-z

**Published:** 2017-08-22

**Authors:** Aneta Spyra

**Affiliations:** Department of Hydrobiology, Faculty of Biology and Environmental Protection, University of Silesia, Bankowa 9, 40-007 Katowice, Poland

**Keywords:** Woodland ponds, Forest, pH, Acidity, Gastropods, Forest landscape

## Abstract

In recent years, the number of areas remaining under the influence of acidity has increased. At all levels of ecosystems, biodiversity decreases with acidification, due to the elimination of species that are most sensitive to low pH. Forest ponds belong to a specific group that varied in location, a huge amount of leaf litter, and isolation from other aquatic environments. They are crucial in the industrial landscape with well-developed industry and human activity. The aim was to investigate the relative importance of water chemistry in explaining snail assemblage compositions and species richness in forest ponds of contrasting pH. Patterns in gastropod communities were determined from an analysis in 26 forest ponds with multivariate gradient analysis. Ponds ranged in a base mean pH from 3.0 to 9.0. pH has been found to be an important factor influencing gastropod fauna. Neutral ponds support diverse communities, typical of small water bodies. In two acidic pond types, snail fauna was different. Among the species characteristic for acidic ponds (pH < 6) were *Anisus spirorbis* and *Aplexa hypnorum.* The greatest distinct characterised alkaline ponds with the numerous appearance of alien *Physa acuta*. The most diverse gastropod fauna was found in neutral ponds, whereas the lowest degree of diversity was found in ponds with the lowest pH. Current knowledge of pH-associated changes in aquatic ecosystems is still incomplete because anthropogenic acidification is a recent phenomenon. It is extremely important in forest habitats, since they react more intensively to climatic factors and are often used in landscape management and planning.

## Introduction

Human impacts have considerably diminished aquatic habitats globally (Dodds et al. 2013). Acidification has adversely affected many freshwaters due to the deposition of acidifying pollutants in Europe (Futter et al. 2014), Asia and North America (Hall et al. 1980; Dale et al. 1985; Freda and Dunson 1986; Dangles et al. 2004). Surface water may be acidic either through the impact from strong inorganic acids from atmospheric deposition or by natural processes of organic acidity, or both. The emission of gaseous pollutants (sulphur and nitrogen oxides) and then their transport over long distances and subsequent deposition in the form of acid precipitation contributes to the acidification of aquatic environments. Acidity may alter the solubility of metals and increase their toxicity because metals in a dissolved state tend to be more harmful in soft water (Hunter 1980; Feely et al. 2012). The acidification of rain-water has been identified as one of a most serious environmental problems of transboundary nature (Singh and Agrawal 2008; Lacoul et al. 2011).

The effects of acidification are known as changes in the structure and functioning of ecosystems, damage to forests and the extinction of aquatic organisms. In naturally acidic environments, anthropogenic acidification can be superimposed on the natural condition, thus exacerbating the acidity (Henriksen 1979; Bishop et al. 2000; Laudon et al. 2001; Moiseenko 2005). The acidity of freshwaters can also be induced, more or less, by the inflow of acidic hot spring water (Fukushima et al. 2004), increased pollution from sulphuric and nitric acids (Hall et al. 1980; Moe et al. 2013) and all of the chemicals and fertilisers that are used in agriculture. It appears that, due to their locations, small water environments that are located in forested areas are particularly vulnerable to acidification (Nisbet and Evans 2014). They appear to be sensitive to continued acid deposition because they depend on rain and snowmelt for most of their water (Cole and Fisher 1979; Fay Baird et al. 1987).

Forest ponds create specific habitats in which the forest locality often influences their physical and chemical conditions. Ponds are known to be elements of a small retention and play a significant role in the water relations of forest areas (Jeffries 1991; Williams 2005). As is clear from previous studies that have been conducted in woodland ponds (Spyra 2010, 2014; Spyra and Strzelec 2014), their characteristic features, among others, are isolation from other water habitats, a small area and depth, significant fluctuations in the water level, variability in the water chemistry and leaf deposits on the bottom. All of these factors mean that the environmental conditions in such ponds are not very stable. Forests and forest management practices can also affect surface water acidification in a number of ways (Nisbet and Evans 2014). According to Korytowski and Szafrański (2008), forest ponds react more intensively to climatic factors than do soils in forest sites that are adjacent to those ponds.

The density of invertebrate declines with a reduction in the pH values (Townsend et al. 1983; Kimmel et al. 1985), and the same situation is observed in relation to the species richness and diversity (Raddum and Fjellheim 1984; Hall and Ide 1987). However, most studies have been carried out in streams and other lotic environments (Rosemond et al. 1992; Guerold et al. 2000; Braukmann 2001; Buffam et al. 2007). Much of the research that has been undertaken on pond acidity in relation to fauna was carried out in connection with the occurrence of amphibians (Dale et al. 1985; Ling et al. 1986; Freda 1986; Freda and Dunson 1986; Albers and Prouty 1987), fish (Schofield 1976; Schofield et al. 1986, 1989; Poleo et al. 1997) and crustaceans (Kawamura et al. 2015). Most of the investigations have either been qualitative field surveys or laboratory experiments on the physiological responses of organisms to lower pH (Hall et al. 1980; Fukushima et al. 2004; Lefcort et al. 2015).

Despite the fact that freshwater snails are a numerous group of benthos and that they perform a key role in the functioning of aquatic ecosystems (Garg et al. 2009), little is known about the influence of pH on their communities in lentic habitats (Lefcort et al. 2015), among them in forest ponds. Because some investigations have found no effect of pH on invertebrate density (e.g. Harriman and Morrison 1982; Simpson et al. 1985; Winterbourn and Collier 1987), this study was designed to examine the influence of forest ponds with contrasting pH on the diversity of freshwater snails in an urban landscape. The objectives were to test the following three hypotheses with regard to freshwater snails exposed to different levels of pH:

1. Does the pH influence the occurrence and diversity of freshwater snails in forest ponds?
2. Is there is a significant interaction between acid, natural and alkaline forest ponds and the composition and spatial variability of snail assemblages?
3. Is the richness and diversity of snails higher in alkaline or in neutral forest ponds?

## Material and methods

### Study area in an industrial landscape

**Fig. 1:**
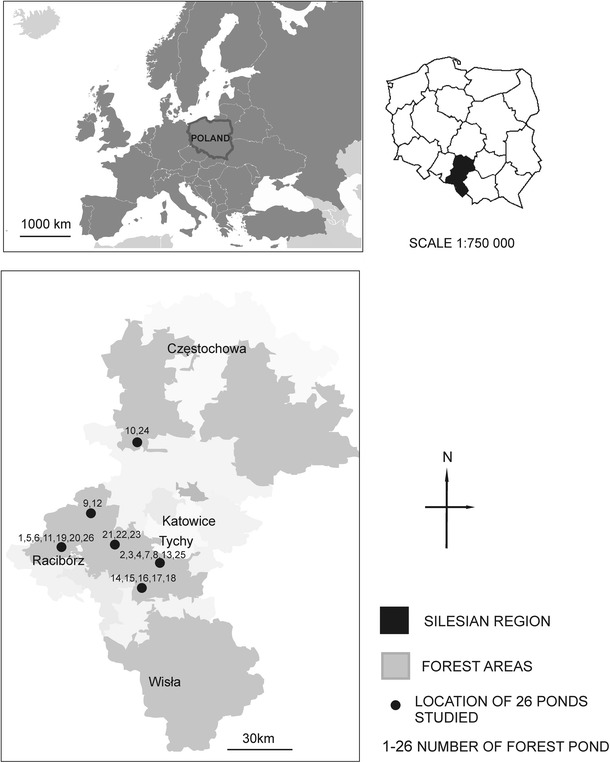
Location of the forest ponds (forest landscape, southern Poland, Silesian Region), 1–26 number of ponds. Geographical coordinates: 1—50° 06′ 59″ N, 18° 33′ 17″ E; 2—50° 12′ 37″ N, 18° 38′ 59″ E; 3—50° 13′ 23″ N, 18° 37′ 19″ E; 4—50° 12′ 32″ N, 18° 37′ 56″ E; 5—50° 07′ 24″ N, 18° 33′ 48″ E; 6—50° 07′ 26″ N, 18° 33′ 60″ E; 7—50° 12′ 34″ N, 18° 39′ 33″ E; 8—50° 11′ 19″ N, 18° 41′ 12″ E; 9—50° 15′ 27″ N, 18° 46′ 17″ E; 10—50° 23′ 45″ N, 18° 34′ 54″ E; 11—50° 07′ 23″ N, 18° 16′ 07″ E; 12—50° 15′ 39″ N 18° 46′ 25″ E; 13—50° 12′ 36″ N, 18° 38′ 00″ E; 14—50° 04′ 09″ N, 18° 59′ 29″ E; 15—50° 04′ 52″ N, 18° 59′ 40″ E; 16—50° 06′ 05″ N, 18° 56′ 16″ E; 17—50° 06′ 17″ N, 18° 56′ 23″ E; 18—50° 05′ 46″ N, 18° 57′ 05″ E; 19—50° 08′ 36″ N, 18° 17′ 14″ E; 20—50° 08′ 06″ N, 18° 17′ 01″ E; 21—50° 10′ 41″ N, 18° 46′ 07″ E; 22—50° 11′ 47″ N, 18° 44′ 12″ E; 23—50° 10′ 36″ N, 18° 46′ 04″ E; 24—50° 23′ 36″ N, 18° 34′ 30″ E; 25—50° 11′ 19″ N; 18° 41′ 00″ E; 26—50° 08′ 48″ N, 18° 18′ 08″ E

The study area comprises a forest landscape neighbouring non-forested areas (Fig. 1). This landscape is largely exposed to strong anthropogenic pressure, which is manifested by changes in water chemistry. Forest ponds constitute parts of the industrial landscapes of the southern part of Poland (50° 15′ N, 18° 28′ E) along with other types of anthropogenic water bodies, heaps and mines. Changes in the landscape followed as a result of well-developed industries, with mainly both surface and underground intensive coal mining are especially noticeable. Ponds were created in the last 90 years when the greatest transformation of the landscape of Southern Poland occurred. Land deformation occurred both during and after coal exploitation, and its consequences are visible after a few years. This process resulted in the formation of new ponds, which constantly enlarge their surface area in random locations (e.g. in the forests, situated near forests and others) (Fig. 1). The forest ponds of this study are similar in area and depth, but their characteristic feature is a wide spectrum of pH. Mud bottom sediments are characteristic for acidic forest ponds, whereas neutral and alkaline ponds have a sandy muddy bottom. Bottom sediments were covered with leaf detritus. The ponds differ in fluctuation in the water level and drainage (Table 1). A few of the ponds are surrounded by a large agglomerations of *sphagnum* sp., which means that their shores are not available and that sampling is difficult; the colour of the water in these ponds is brownish.

**Table 1:** Acidic, neutral and alkaline forest ponds characteristics, range of variation (min-max) in relation to the eudominant class of snails, pH- in average

	Characteristic features		Eudominant snails	pH	Type of pond
Age Area (ha) Mean depth (m)	1970–1993 1.2–12.3 1.5–3.0	Very high fluctuations in water level drying out: the length of dry periods—from 2 weeks to 1 month mud	*R. balthica* *P. planorbis*	5.0	Acidic < 6 (pH 3.0–5.9)
Age Area (ha) Mean depth (m)	1920–1991 2.02–34.9 1.5–3.0	High fluctuation in water level not drying out mud	*H. complanatus* *A. vortex* *B. contortus*	6.2	Acidic ≥ 6 (pH 6.0–6.7)
Age Area (ha) Mean depth (m)	1929–1979 0.5–14.4 1.2–3.2	High fluctuations in water level not drying out sand + mud	*P. planorbis* *A. vortex* *B. contortus*	7.0	Neutral (pH 6.8–7.2)
Age Area (ha) Mean depth (m)	1926–1974 2.6–29.0 1.5–4.0	Small fluctuation in water level not drying out sand-mud	*P. acuta*	8.5	Alkaline (pH > 7.2)

### Field surveys and laboratory analysis

The research was conducted in 26 forest ponds with contrasting pH. Gastropod were sampled three times in each pond (Spring, Summer and Autumn, Brewin et al. 1996; Bishop et al. 2000) using the methods of quantitative collection (square frame, sampling area 0.5 m^2^). In each pond, material was taken from the allochthonic matter (detritus deposits). In the laboratory, the samples were washed and sifted through a grade 0.02 mesh size sieve. They were sorted under a stereoscope microscope. The collected organisms were fixed in 75% ethanol and were identified according to the standard keys provided by Piechocki (1979), Gloer (2002) and Piechocki and Wawrzyniak-Wydrowiska (2016). The numbers of each species (density) are expressed as individuals/square meter.

In the ponds with a contrasting pH, water samples were collected for physico-chemical analyses. Some of the parameters, that is, water temperature, pH, conductivity and TDS, were measured in the field using an electrode.

The faunistic similarities of the freshwater snail communities in the four types of forest ponds were estimated using an analysis of the hierarchical clustering method (Statistica Software ver.12). Because of the ecological context of this study, the hierarchical clustering analysis has been realised using the species dominance data as the basis for clusterisation. This method takes into account the relative rareness and commonness of species that may be affected by the pH. Ward’s method was used as the linkage rule and the Euclidean distances as the distance measure.

The pH ranges for the various types of forest ponds were adopted according to Økland (1990): acidic 4.4–6.6 (ponds 1–12), neutral pH 6.8–7.2 (ponds 13–20) and alkaline pH > 7.2 (ponds 21–26). A similar classification was used by Kabisch and Hemmerling (1982), but they also distinguished a group of extremely acidic ponds with pH values of 1.8–4.5. Since only two ponds in this study fit into such a group of ponds, having such a category appeared to be unjustified. In the literature, the group of “extremely acidic” ponds include ponds with water of varied pH values, e.g. pH < 4.5 (Schofield 1976), pH ≤ 3.6 (Nordstrom et al. 2000), 1.8–2.8 (Havas and Hutchinson 2011). In order to determine the composition of the snail assemblages in the ponds in which the pH values were below 6.0 in this research, “acidic ponds” were divided into two groups depending on the pH values: pH < 6.0 and pH ≥ 6.0 (6.0–6.7).

Several indices were used to characterise the snail communities in the ponds of contrasting pH as follows: dominance (D) (%) was calculated according to Zawal et al. (2013) eudominants > 10.0%, dominants 5.01–10.0%, subdominants 1.01–5.0%, recedents 0.51–1.00 and subrecedents < 0.50%. Frequency (F) (%) was adopted according to Górny and Grüm (1981): constant species (100–75.1%), common species (75–50.1%), rare species (50–25.1%) and accidental species (≤ 25%). The Shannon-Weiner and Simpson biodiversity indices were used to examine the diversity of snail communities in the forest ponds (MVSP 3.13.p Kovach Computing Services).

### Statistical analysis

In order to determine how the environmental variables relate to one another and to determine the overall temporal and spatial influences on the snail populations, a unimodal response model of multivariate ordination analysis was used (Lepš and Šmilauer 2003). The DCA gradient length produced from the Gastropod data set was 5.2 (SD); therefore, the Canonical Correspondence Analysis (CCA) (direct gradient analysis) was used to explore the relationship between the snail communities and the environmental variables (CANOCO 4.5). The results of the Forward Selection showed that, among the 27 environmental variables taken into account in the analysis (water chemistry, bottom sediments, a fluctuations in water level, drainage of ponds, their size, age, depth and distance between ponds), 11 were statistically significant. The statistical significance of the environmental variables in the model and the relationship between them and the biological data was evaluated using the Monte Carlo permutation test (499 permutations). CANODRAW (ver.4.5) was used to create an ordination diagram that shows the patterns of the variation in community composition that can be best explained by the environmental variables. According to Ter Braak (1986), such a diagram approximately visualises the “centres” of the different species distributions along each of the environmental variables.

The significance of the differences in the values of the environmental variables, the diversity indices and the density of gastropods between the ponds was evaluated using the ANOVA Kruskal-Wallis and multiple comparisons post hoc test. The data was found to be of a non-normal distribution (Kolmogorov-Smirnov test for normality) (STATISTICA 12.0)

## Results

### Patterns in the species richness and composition of the snail assemblages in acidic, neutral and alkaline forest ponds

A total of 22 species of freshwater snails were found in the forest ponds of contrasting pH (Table 2). Only 8 species occurred in ponds with the lowest pH (from 2 to 6 species in specific ponds), and 18 species were found in neutral and acidic ponds (pH ≥ 6.0) (4–12 and 3–10, respectively).

**Table 2:** Dominance patterns and mean density (ind./m^2^) in acidic, neutral and alkaline forest ponds

No of ponds	Ponds 1–4		Ponds 5–12		Ponds 13–20		Ponds 21–26	
Type	Acidic < 6		Acidic ≥ 6		Neutral		Alkaline	
	D (%)	Density	D (%)	Density	D (%)	Density	D (%)	Density
*B. tentaculata*	0.0	0	0.5	7	0.5	8	0.0	0
*P. acuta*	0.0	0	0.0	0	0.0	0	81.6	954
*R. balthica*	55.3	58	1.4	21	3.6	59	3.1	36
*R. auricularia*	0.0	0	2.9	43	0.0	0	1.4	16
*L. stagnalis*	0.0	0	1.0	15	0.1	1	0.5	5
*P. planorbis*	13.8	14	5.9	87	13.2	220	1.4	17
*S. nitida*	0.0	0	4.1	61	3.5	59	0.0	0
*G. crista*	9.1	10	2.8	41	7.2	119	0.7	8
*G. albus*	6.1	6	2.4	35	1.2	20	0.0	0
*P. corneus*	9.1	10	5.2	77	3.4	57	0.4	4
*H. complanatus*	0.0	0	21.1	313	6.8	112	0.0	0
*A. vortex*	0.0	0	14.7	218	23.6	393	3.1	36
*F. fragilis*	0.0	0	1.2	18	3.0	50	1.9	23
*S. palustris*	0.0	0	0.1	2	0.2	3	0.3	4
*S. corvus*	1.6	2	0.4	6	0.6	10	0.0	0
*B. contortus*	0.0	0	35.3	523	30.7	512	1.7	20
*P. antipodarum*	0.0	0	0.0	0	0.3	4	0.6	7
*A. spirorbis*	2.2	2	0.1	1	0.0	0	0.0	0
*A. hypnorum*	2.8	3	0.0	0	0.0	0	0.0	0
*P. fontinalis*	0.0	0	0.4	6	0.6	9	1.9	22
*G. truncatula*	0.0	0	0.0	0	0.1	2	1.2	14
*V. cristata*	0,0	0	0.4	6	1.5	24	0.0	0
*N*	1274		35,532		40,042		21,042	
Density: mean min-max	106 15–256		1480 156–4042		1668 228–7157		1169 180–4269	

*N* number of collected specimens

**Fig. 2:**
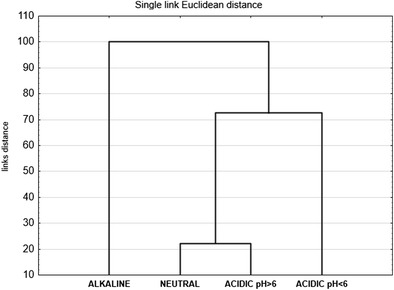
The results of the analysis of the hierarchical clustering method in the forest ponds

The results of the analysis of the hierarchical clustering method indicated that the compositions of the snail communities in the four types of ponds were grouped in relation to pH (Fig. 2). Alkaline ponds with only one species that occurred numerously—*Physa acuta* (eudominant)—were distinctly characteristic. The highest values of pH were observed in ponds 21 and 22 (pH 7.7–9.2 and 8.0–9.0, respectively).

The other ponds belonged to the second group (Fig. 2). A quite similar community structure appeared to characterise the neutral and acidic ponds with pH ≥ 6 due to the numerous occurrence of *Bathyomphalus contortus* and *Anisus vortex*; however, in neutral ponds, *Planorbis planorbis* also belonged to eudominants, whereas, in acidic ponds (pH ≥ 6) it was *Hippeutis complanatus*. In both groups of ponds, 18 species of snails occurred, which had an impact on the results of the clustering layout. The faunistic analysis proved that the distinction of the two groups of forest ponds within the acidic ponds was justified. The structure of the snail communities was clearly distinct in this group of ponds with two eudominant species: *Radix balthica* and *P. planorbis* (Table 2). In pond 2, in which the pH values ranged from 5.0 to 5.5, only 2 species *R. balthica* and *P. planorbis* were found but in very small number. Their mussels were very thin and translucent. A similar situation was found in pond 1 but *Gyraulus albus* was also collected. In the other ponds belonging to this group (pond 3 and 4), pH ranged from 4.0–6.0 and 3.0–6.0, respectively, and six species of snail were found in both of them.

**Fig. 3:**
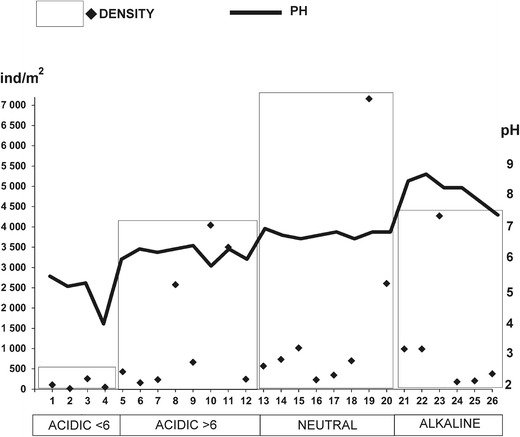
Average density of freshwater snails in the forest ponds of contrasting pH

The density of snails was clearly related to pH, and the lowest (mean 106 ind/m^2^) were found in acidic ponds with pH < 6.0 (ANOVA, Kruskal-Wallis H (4.*N* = 19.776, *p* = 0.0002; post hoc: *p* = 0.0001), whereas the highest was found in neutral ponds (mean 1668 ind./m^2^) (Table 2). It is visible from Fig. 3 that the lowest densities were found in ponds 2 and 4 in which the lowest mean pH values were found. In the acidic ponds with pH ≥ 6, the highest densities of snails were found in ponds 8, 10 and 11, and this was related to the numerous occurrences of *H. complanatus* in pond 8, and *B. contortus* in ponds 10 and 11. In the alkaline ponds, the highest densities were observed in the ponds where *P. acuta* numerously occurred (Fig. 3).

### Snail assemblages in the forest ponds with contrasting pH in relation to the other properties of the water

The water in the four groups of forest ponds studied differed in the values of pH, and these results were statistically significant (ANOVA, Kruskal-Wallis, H (3.*N* = 63.3122, *p* = 0.00001, post hoc: *p* = 0000.1). As is clear from the analysis of water chemistry, forest ponds were different in terms of their salinity and biogenic properties. The highest values of conductivity (5900 μS/cm), TDS (2940 mg/l) and chlorides (930 mg/l) were found in the alkaline ponds. The differences in those parameters between the forest ponds were statistically significant (Table 3). The concentrations of nitrate, nitrite, ammonia, phosphates, calcium and total hardness were very high in those ponds. In the neutral ponds, the values of all the parameters studied were not very high except for nitrates (the highest value was 20.8 mg/l) (Table 3). In the acidic ponds with pH < 6, high values of conductivity, TDS, chlorides, nitrates and iron were also observed. The values of total hardness and calcium content were very different in comparison to other group of acidic ponds (Table 3).

**Table 3:** The physico-chemical characteristics of the water in ponds of contrasting pH

Temp. °C	Conduct. μS/cm	TDS mg/l	Cl mg/l	NO3 mg/l	NO2 mg/l	NH3 mg/l	PO4 mg/l	T. hard. mgCaCO_3_/l	Ca mg/l	Fe mg/l	Type of pond
6.0–20.3	200–4140	60–2090	15–895	0.0–50.0	0.002–0.07	0.05–8.0	0.0–0.52	71–789.4	0.0–190	0.3–25.6	Acidic < 6
7.0–28	140–750	75–370	16–86	0.0–29	0.0–0.8	0.05–25	0.2–1.0	71.4–357	15–81	0.35–5.41	Acidic ≥ 6
6.0–24.5	80–1420	30–670	6.0–189	0.89–20.8	0.01–0.3	0.03–7.0	0.0–0.5	39–451	18–140	0.63–3.99	Neutral
8.0–23.6	390–5900	190–2940	61–930	0.0–45.2	0.0–0.13	0.09–39	0.03–2.46	98–2465	45–314	0.13–6.5	Alkaline
	34.099* *p* = 0.0001	33.943* *p* = 0.0001	33.695* *p* = 0.0001	7.252 *p* = 0.064	6.394 *p* = 0.093	16.153* *p* = 0.004	10.369* *p* = 0.015	11.201* *p* = 0.0107	22.190* *p* = 0.0001	14.887* *p* = 0.002	ANOVA H (3.*N* = 78)

*Temp.* temperature, *Conduct*. conductivity, *T. hard.* total hardness

*Statistically significant results

**Fig. 4:**
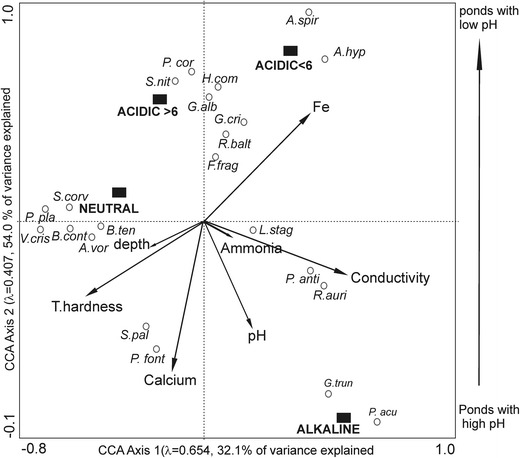
CCA species biplot based on the snail data and statistically significant environmental variables

The results of the CCA analysis (Table 4, Fig. 4) showed that snail composition varied in the gradient of different pH. The group of species that were characteristic to acidic ponds with pH < 6 primarily included *Anisus spirorbis* and *Aplexa hypnorum.* Most of the gastropods were associated with the neutral ponds, e.g. *P. planorbis*, *Valvata cristata*, *B. contortus*, *A. vortex*, and *B. tentaculata*. The occurrence of a few of them was also influenced by high values of total hardness and the depth of the water. Depth was quite similar in forest ponds of this study except the alkaline ponds in which the depth was the greatest. *G. truncatula* and *P. acuta* were associated with the alkaline forest ponds (Fig. 4). *Potamopyrgus antipodarum* and *Radix auricularia* mainly occurred in the waters that had values of conductivity and pH, whereas the occurrence of *Lymnaea stagnalis* was strongly influenced by high values of ammonia.

**Table 4:** Summary of CCA analysis based on the species and environmental data in acidic, neutral and alkaline forest ponds constrained by 27 environmental variables

CCA axes		1	2	3	4	Total variance
Eigenvalues		0.654	0.407	0.264	0.183	3.509
Species-environment correlations		0.937	0.905	0.764	0.726	
Cumulative (%) variance of species data of species-environment relation						
		15.8	287.5	34.9	40.1	
		32.1	54.0	68.8	79.0	
Sum of all eigenvalues						3.509
Sum of all canonical eigenvalues						1.782
Monte Carlo significance	λ1	*F* ratio	*p* value		Trace	Permutations
First axis All axes	0.917	10.416 3.256	0.0020 0.0020		3.256	499 499

These results are consistent with the values of the diversity indices. The lowest average Shannon Weiner diversity values were found in the alkaline ponds (0.59) and in the acidic ponds with pH < 6 (0.67) (ANOVA, Kruskal-Wallis, H(3.*N* = 13.71, *p* = 0.003; post hoc: *p* = 0.001). The maximum diversity was found in the neutral ponds (1.08). The values of the Simpson diversity, similar to the Shannon index, was found to be the lowest in the alkaline and acidic (pH < 6) ponds (0.325 and 0.380, respectively) and the highest in the neutral ponds (0.55) (ANOVA, Kruskal-Wallis H (3.*N* = 9.25, *p* = 0.0002; post hoc: *p* = 0.0001).

## Discussion

### Patterns in the species richness and composition of the snail assemblages in acidic, neutral and alkaline forest ponds

Aquatic environments are affected by a variety of variables that can be correlated with the gastropod community. This study involved 26 forest ponds of contrasting pH and showed that they differed statistically in pH values. It is impossible to perform a study on pH influence on gastropods in ponds that differ only in terms of pH even in ideal conditions because of the direct and indirect influences of pH on physical environments. In this study, the impact of the substrate type was partially reduced due to sampling snails from leaf deposits as a basic substrate, characteristic to forest ponds. Others factors, e.g. pond size, age, distance from other ponds, fluctuation in the water levels and pond drainage, were not statistically significant. The data on some variables that may influence the snail communities, e.g. dissolved oxygen content, turbidity and food availability in each site, were not available.

Classifications of acidic waters (Driscoll et al. 1989; Futter et al. 2014) include critically acidic habitats with pH < 4.5 (Schofield 1976) or extremely acidic with pH 1.8–2.8 (Kleeberg 1998; Havas and Hutchinson 2011) also with pH < 0.0 (Nordstrom et al. 2000). A few ponds of this study are constantly acidic (e.g. ponds 1, 2 and 4) and other ponds’ acidity is episodic, probably due to annual desiccation, in which very low pH values appears after the end of a dry period. In intensively drained wetlands (Johnston et al. 2014), the pH values range from 3 to 4, whereas in permanently wet areas, it varied from 5 to 6.5 and this is consistent with this study. The pH can fluctuate within daily and seasonal timeframes (Tucker and D’Abramo 2008); snails can, however, become stressed or die when exposed to pH extremes or when pH changes rapidly, even if the change occurs within a pH range that is normally tolerated.

Coniferous trees provide a huge allochthonous pulse inflow in the form of conifer pines in the water along the shoreline of some ponds of this study (e.g. ponds 2, 5, 6 and 12). The presence of *sphagnum* around them appears to be linked to the low pH especially since surface run-off may be responsible for transferring important nutrient loads into water bodies. Klimaszyk and Rzymski (2011) observed a brown colour and low pH in forest lakes in periods with the highest rainfall and surface runoffs, which indicates their significant role in determining of dystrophic conditions. *Sphagnum* moss and filamentous algae are acid tolerant, and become large in pH below 5.5 (Singh and Agrawal 2008). The results of this study indicate that it is likely that the pH can be a good indicator of different chemical regimes and affects the diversity of snail assemblages. In the alkaline ponds, the diversity was lower than in the natural ponds. In the acidic ponds, the diversity was lower than in ponds with higher pH and these results were statistically significant. In the past few decades, it has become increasingly clear that acidification negatively affects the ecosystem function and diversity (Guerold et al. 2000; Braukmann 2001) due to the elimination of species that are most sensitive to low water pH (Moiseenko 2005), also in relation to gastropods (Økland 1990). The effects of anthropogenic acidification on the snail assemblages seems to be more severe than those of natural acidity (Jüttner et al. 1997), which must have been present much longer (Dangles et al. 2004; Petrin et al. 2007). In addition to the study of Dangles et al. (2004), it is known that its strong effect may be a consequence of a low capacity to adjust to rapidly changing conditions in drying ponds such as in this study.

Unless pH strongly determined species composition (Petrin et al. 2007), low pH is believed to lead to impoverished communities (Heino 2000), also in the relations to snail species richness (Hoverman et al. 2011). However, some research negates the fact that there is a lack of gastropods in acidic environments, e.g. Garg et al. (2009) found that molluscs are independent of fluctuations with respect to the pH. In this study in pond 3 in which pH only periodically decreased to an extremely low value, the diversity of snail was higher than in the other ponds within this group (the average density 256 ind/m^2^), and in ponds 1, 2 and 4 the occurrence of snails were found only in a small numbers. The study of Økland (1992) focused on the ecological aspects of the Norwegian acid lakes found that acidity is the main reason why snails are absent in a low-calcium area that is influenced by acidic precipitation, and they were not detected below pH 5.2. In this research, *A. spirorbis* and *A. hypnorum* mainly belonged to the species that were characteristic of acidic ponds with pH < 6. They are known as tolerant to desiccation (Kerney 1999; Piechocki and Wawrzyniak-Wydrowiska 2016). Lefcort et al. (2015) found that the response of snails to rising anthropogenic carbon dioxide levels may be more complex than was once believed. What they found was that snails seem to be preadapted to a low pH. Their short-term survival in experimental acidic conditions was better than snails from reference sites that had no metal pollution. The survival of the snails from the polluted sites was very high. The mechanism of this phenomenon still remains unknown but Sullivan and Cheng (1975) and Lefcort et al. (2015) indicated that it may be connected with an altered epithelium membrane permeability or an altered mucus secretion.

Hoverman et al. (2011) found that species richness was positively correlated to the area, hydroperiod and pH of the water. Thus, habitats that have a high snail diversity tended to be large, permanent and with alkaline conditions. This is only partially consistent with this study because a relatively low diversity was found in alkaline ponds, which resulted from a mass occurrence of *P. acuta*. As previously indicated (Spyra and Strzelec 2014), it occurs within a wide spectrum of pH (6.0–9.0); however, in this study, it only was only found in ponds with the highest pH (7.3–9.2). It is obvious that snail species have different tolerance ranges at various levels of pH. According to the findings of Schindler (1988), Økland (1992) and Singh and Agrawal (2008), the number of snails and phytoplankton fell below pH 5.5, while snails and zooplankton disappeared in pH less than 5.2. There are no precise guidelines for a high pH tolerance, but pH values above 9.5 or 10 are generally considered to be undesirable in ponds (Tucker and D’Abramo 2008). This study showed that, in the alkaline ponds, 14 species of snails were found, although 13 of them in rather small numbers and only two species were associated with these ponds (*P. acuta* and *G. truncatula*). According to Bishop et al. (2000), while higher pH promotes certain species, the ability to survive the stress associated with a natural episodic pH decrease is a competitive advantage for other species.

Changes in pH can result in variability in the food supply, which can indirectly influence survival. Studies have documented changes in detrital (Friberg et al. 1980), algal (Hall et al. 1980) and food affluence with changes in pH values. In the study of Hall et al. (1980), acidification decreased species diversity, increased the representation of dominants and decreased the complexity of the food web. The periphyton biomass and basidiomycete fungus increased at low pH, while hyphomycete fungal densities decreased. The influences of various factors on the occurrence of freshwater snails are complicated and not easy to explain because their impacts are linked. Folt et al. (1999) noted that natural and anthropogenic factors can act in combination to create effects that are greater than the effect of each factor individually. The fact is that pH is likely to influence the water chemistry and the composition of snail fauna. Garg et al. (2009) found that the richness of molluscs may be attributed to the cumulative effect of a high calcium content, alkaline nature of water and macrophytes.

### Snail assemblages in the forest ponds with contrasting pH in relation to the other properties of the water and leaf deposits

The water chemistry and trophic conditions is of special importance (Friday 1987; Jeffries 1991), and in ponds that have extreme water chemistry, a decrease in snail species richness have often been documented. The water parameters related to acidity have often been cited as important factors in lentic ecosystems (Rasmussen and Kalff 1987; Brodersen et al. 1998). The impact of acidification manifests itself within a short period of time due to the high degree of sensitivity of molluscs, crustaceans or aquatic insects (Yakovlev 1998; Raddum and Skjelkvale 2001). Forest ponds of this study differed significantly in water chemistry. In the study of Hall et al. (1980), concentrations of, e.g. Ca, Mg, Mn and Fe were elevated with increased water acidity, and no change in NO_3_ and NH_4_ occurred at lower pH. In forest ponds of this study, the highest ammonia content was characteristic of the alkaline ponds (39 mg/l). The highest concentration of nitrates was found in ponds with the lowest pH (50 mg/l) as well as in the alkaline ponds (45.2 mg/l). Woodland ponds have a relatively high content of Fe (Spyra 2014). Its highest values were found in the acidic ponds (pH < 6.0, 25.6 mg/L). In contrast, in neutral ponds, the highest value of Fe was 3.99 mg/l, and in acidic ponds of pH ≥ 6, it was 5.41 mg/l. A high content of Fe significantly influenced the occurrence of *Gyraulus crista*, *R. balthica*, *A. hypnorum* and *A. spirorbis*. By indicating the changes in water chemistry in extremely acidic to neutral mining ponds over time, Kleeberg (1998) stated that the concentration of Fe is quite variable and that the Fe concentration in acidic ponds (pH < 6) is 170 times higher than that of ponds with pH > 6 and 7 to 9 times higher than that of the neutral ponds.

The CCA analysis combined the physical, chemical and biological data and permitted a summary of the key patterns in the variation in the data to be created. Although the content of biogenic substances was high in the alkaline ponds, CCA showed no statistical importance of these variables in shaping the composition of the snail communities. *Stagnicola palustris* and *Physa fontinalis* were associated with the high content of calcium. Both species occur in alkaline ponds. In waters that were rich in calcium content, Dutta and Malhotra (1986) found a predominance of snails in a fish pond at Jammu. Acid rain results in the leaching of Ca ions; therefore, it disturbed the process of shell formation in molluscs, which are more susceptible to acidification. Roff and Kwiatkowski (1977) found no snails in Ontario lakes in pH at or below 5. In this study, the conductivity and total hardness were found to be statistically important. An increase in the total hardness of water favours the growth of molluscs and shows a positive correlation to the mollusc in the study of Garg et al. (2009).

The formation of leaf deposits is a typical feature that distinguishes forest ponds from other aquatic environments (Oertli 1993; Dangles et al. 2001; Spyra 2010, 2014). The input of allochthonous matter affects the stability of the food webs, which depend on the quality and the size of the incoming pulse and the time at which it appears in a pond. A moderate and low input stabilise the relations in a trophic system, while an input that is too high has a destabilising effect (Oertli 1993). The decay of leaf litter depends on, e.g. the species of trees, the depth and the presence of shredders. It also seems that it depends on acidity. According to the results obtained by Griffith and Perry (1993), leaf litter processing rates are the fastest in neutral streams, slowest in acidic stream and intermediate in the most alkaline. However, Singh and Agrawal (2008) found that the decomposition rate in acidified lakes is slowed down because the fungi and bacteria are not tolerant of acidic conditions. In the case of the forest ponds studied here, it is hard to indicate whether the size of the leaf deposit are associated with the selected types of ponds because in three of the four acidic ponds (pH < 6) the layer of leaf deposit was thick. In three of the eight studied acidic ponds (pH ≥ 6), the layer of leaf deposits was thin. In all alkaline ponds, the layer of leaf deposits was also thin. Dangles et al. (2004) showed that the taxonomic richness and the breakdown rate of leaf litter in naturally acid streams were not significantly different from the richness and breakdown rates of three neutral control streams in the same area in northern Sweden.

Water acidification is a complex process. Investigations dealing with the effects of acidification on freshwater fauna have been performed in the Scandinavian countries, America, Canada and Russia, where this problem has received special attention. The pattern of low pH has been found in temporary ponds across North-eastern North America (Dale et al. 1985; Freda and Dunson 1986; Ling et al. 1986; Albers and Prouty 1987). However, in Poland to date, the ecological consequences of water acidification have not been assessed comprehensively. This study emphasises the importance of considering multiple stressor interactions in hydrobiological and ecological research. In recent years, the number of areas that remain under the influence of acidity has increased. Many invertebrates are sensitive to acidification, with some disappearing at pH values as high as 6.0 (Schindler 1988). This is not always consistent in relations to freshwater snails. An abrupt drop in pH in streams during spring and rain floods, which is referred to as pH shock, is especially dangerous for biological life (Moiseenko 2005), but low pH as a consequence of summer drying up or the inflow of humus substances also appears to be dangerous. According to CCA results among the different parameters of the water, it is likely that pH has effects on snail populations. The species composition and relative species’ abundances in communities are affected by the general chemical regime of a water body arising as a cumulative result of many factors. As is clear from this research, pH (especially its low and high values) is one of the important factors that influence the specific water chemistry regime in ponds of this study. In this context, pH can be indicated to be one of the main drivers of the formation of snail communities in forest ponds. The current knowledge of pH-associated changes in aquatic ecosystems may help us to better understand what the real impact of pH on gastropod diversity is because they are key players in many freshwater food webs.
